# Undiagnosed apical hypertrophic cardiomyopathy in an old amateur soccer player: a case report

**DOI:** 10.11604/pamj.2021.40.182.28588

**Published:** 2021-11-25

**Authors:** Mahassine El Harras, Ilham Bensahi, Salma Abdeladim, Fatimazahra Merzouk, Amal Elouarradi, Sara Oualim, Mohamed Sabry

**Affiliations:** 1 Department of Cardiology, Mohammed VI University of Health Sciences Cheikh Khalifa Hospital, Casablanca, Morocco

**Keywords:** Hypertrophic cardiomyopathy, apical hypertrophic cardiomyopathy, sport cardiology, case report

## Abstract

Hypertrophic cardiomyopathy is a primary muscle disorder characterized by an abnormal thickness of the left ventricular wall. It is often going undiagnosed because many patients have few symptoms and can lead normal lives. This is a case report about an apical cardiomyopathy diagnosed at a very late stage in an old amateur soccer player. He was hospitalized due to acute chest pain; neurologic disorder related to a hypertensive emergency, he underwent successful percutaneous coronary intervention, echocardiography and CMR revealed Apical hypertrophic cardiomyopathy. The development of sports cardiology has major importance in the detection of cardiac disease which may have poor prognosis. Our patient had the chance to achieve his entire career without rhythmic complications.

## Introduction

Hypertrophic cardiomyopathy is a muscle disorder with several morphological manifestations, characterized by a natural history, and specific prognosis, it´s a complex, primary, and inherited cardiac disease [1,2]. Patients with Hypertrophic cardiomyopathy (HCM) can present one or more of non-pathognomonic symptoms from shortness of breath to arrythmias causing sudden death, hence the interest of early diagnosis, good management and family screening. We report a case of an apical cardiomyopathy none diagnosed in an old amateur soccer player.

## Patient and observation

**Patient information:** a 72-year-old man, high level athlete who exercised as an amateur soccer player during 12 years, with a 1-year history of hypertension, diabetes mellitus since 2010.

**Clinical findings:** he was hospitalized due to a hypertensive emergency.

**Timeline of current episode:** the patient presented an acute chest pain, neurologic disorder, a left sided weakness related to a hypertensive emergency.

**Figure 1:**
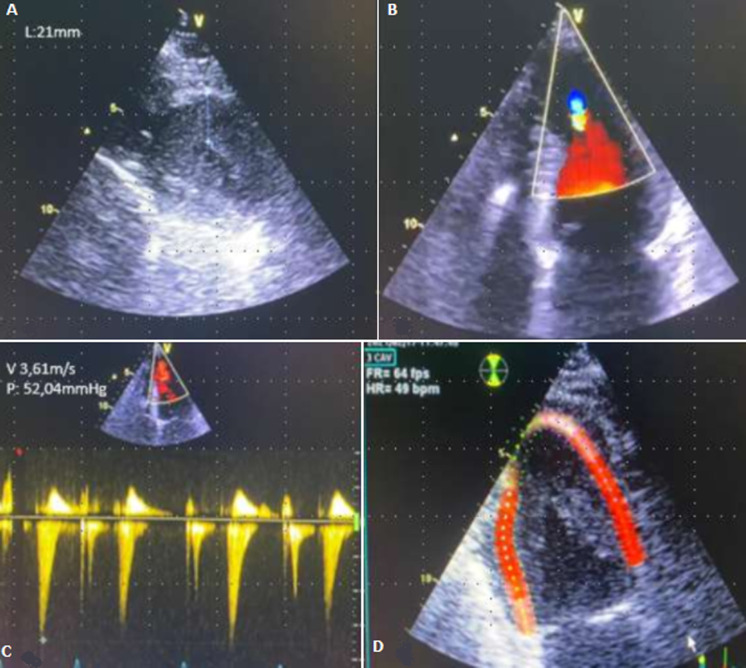
transthoracic echocardiography; A) apical hypertrophic cardiomyopathy with a wall thickness up to 21mm; B, C) left ventricular obstruction; D) altered apical strain

**Figure 2:**
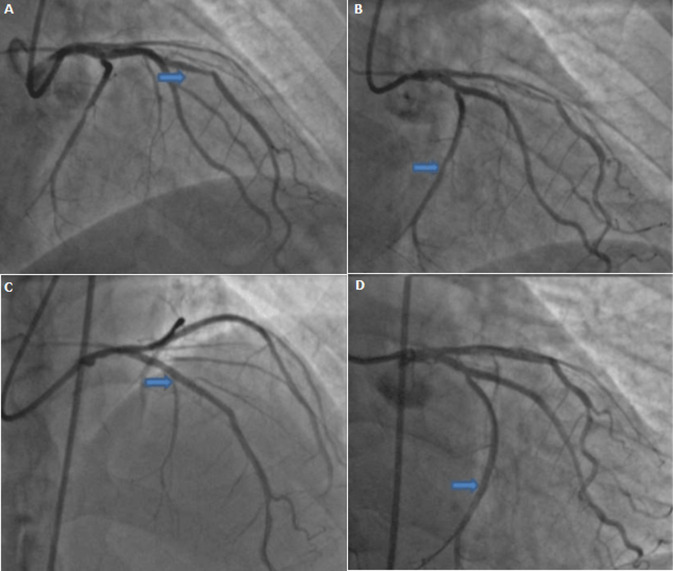
coronarography; A, B) double stenosis in the proximal and middle left anterior descending artery, severe stenosis in the distal circumflex artery; C, D) successful percutaneous coronary intervention interesting left anterior descending artery and circumflex artery

**Figure 3:**
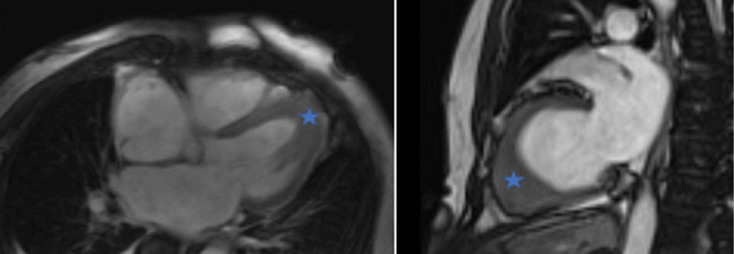
cardiac MRI demonstrating focal apical left ventricular hypertrophy (star), confirming the diagnosis of apical hypertrophic cardiomyopathy

**Diagnostic assessment:** he presented a high blood pressure up to 220/110 mmHg, the electrocardiogram showed an atrial fibrillation with deep negative T waves. Cerebral Magnetic resonance imaging (MRI) revealed a recent left sylvien stroke. Further, echocardiography (Figure 1) revealed normal systolic function with left ventricular ejection fraction (LVEF) as 65%, apical hypertrophic cardiomyopathy the wall thickness was up to 21mm with left ventricular obstruction, he underwent a coronarography, it revealed a double stenosis in the proximal and middle left anterior descending artery, and sever stenosis in the distal circumflex artery, he was taken for successful percutaneous coronary intervention (PCI) through femoral route (Figure 2). Cardiac magnetic resonance imaging (CMR) was performed, it detected apical LV hypertrophy up to 17mm in the apical anterior wall, 12mm in the apical inferior wall, 18mm in the apical septal and lateral walls, with fibrosis in the apex and apical segment of the LV (Figure 3). The Holter electrocardiogram (EKG) showed sustained ventricular tachycardia. The results were consistent with an apical hypertrophic cardiomyopathy corresponding to the fourth type of Marron classification.

**Therapeutic interventions:** the patient was treated with propranolol, ischemic cardiopathy medication and anticoagulant.

**Follow-up and outcome of interventions:** the sustained ventricular tachycardia persisted under medical treatment, according to the European society of cardiology (ESC) recommendation the risk of cardiac sudden death was up to 7.74%, an International classification of diseases (ICD) was placed. A family screening was performed, including medical history, clinical examination, EKG, and a transthoracic echography in the first-degree relatives.

**Patient perspective:** the patient hopes to lead a normal life, and hopes his descendants are free from the disease.

**Informed consent:** the patient was informed, and consent was obtained.

## Discussion

Hypertrophic cardiomyopathy is defined by “the presence of increased left ventricular wall thickness that is not explained by abnormal loading conditions” [3]. Maron described four types, type I corresponds to the basal septum´s hypertrophy, type II corresponds to the whole septum´s hypertrophy, type III the hypertrophy interest the septum, anterior and anterolateral wall and type IV is represented by a LV apical hypertrophy [4]. This fourth type is a rare morphologic variant and an atypical form of HCM along with mid-ventricular obstruction, appointed as Apical HCM, spade shaped, Japanese variety, Yamaguchi syndrome [3,5]. Sakamoto described a cardiac disorder manifested by negative T-waves on electrocardiography, associated with apical hypertrophy of the left ventricle [6]. The prevalence is high in Japan and Asian countries. It´s around 15% in the Japanese population as compared to 3% of a United states cohort [7]. Apical HCM has been recognized as familial disease, implicating the role of genetics in the development of this morphological pattern of hypertrophy [8]. More than half of patients are asymptomatic, they are male and are diagnosed during their 4^th^ decade [9]. The most common symptom is atypical chest pain, dyspnea, exercise intolerance, palpitations, atrial fibrillation and syncope [10]. The wave inversions in precordial leads are present in 93% of the cases, the giant and deep T waves are present only in 47%. Furthermore, electrocardiographic left ventricular hypertrophy is found in 65% of the cases [6,11]. The Holter EKG can show the presence of ventricular ectopies, which is a risk factor of sudden death [12]. Typical echocardiographic finding includes increased thickness of the left ventricular wall in the apical region. The cut-off value is fixed at 15mm although in the case of typical clinical symptoms and apex morphology, the wall thickness value is between 13 and 15mm. In the four-chamber view the echocardiographic shape of the left ventricle is compared to an “ace of spades”. The echocardiographic contrast is used to improve the visualization of left ventricle´s morphology and objectify the presence of apical aneurysm [10,13]. The diastolic gradient must be determined; it´s a potential additional risk factor of sudden death [14]. Cardiovascular magnetic resonance (CMR) provide a high-resolution image, complete covering of left ventricle morphology including the apex. It shows a typical “ace-of-spades” silhouette of LV, apex wall width > 15mm with basal/apex wall thickness ratio > 1.5 [15].

The extent of late gadolinium enhancement can be used to predict cardiovascular mortality, but current data do not support its use in prediction of Sickle cell disease (SCD) risk [16]. Making the difference between hypertensive heart disease and HCM is a real challenge, there is some clinical features favoring HCM with systemic hypertension such as family history of HCM, marked repolarization abnormalities, conduction disease or Q-waves on EKG, right ventricular hypertrophy, severe diastolic dysfunction, late gadolinium enhancement, maximum LV wall thickness [3]. The use of a β-blockers or calcium channel blockers is recommended to prolong diastole time in patients with preserved ejection fraction. In patients with reduced ejection fraction typical heart failure medication should be used. All patients with atrial fibrillation should receive anticoagulation Therapy. Surgical myotomy and alcohol ablation are the recommended method of treatment when patients are refractory to optimal medical treatment. The aim of surgical treatment is to increase the volume of the left ventricle [10]. Data about the use of surgical myotomy or alcohol septal ablation in AHCM are limited; a few cases have been reported [17]. The Association for healthcare communications and marketing (AHCM) prognosis is better than other form of HCM, cardiovascular mortality is up to 1.9%, the most frequent morbid event are atrial fibrillation and myocardial infarction [11]. Recent studies suggest that the risk of sudden cardiac death, morbid events, is com¬parable to patients with other variants of hypertrophic cardiomyopathy [18]. According to American guidelines patients who have one of the following risk factors as family history of SCD, syncope, asymptomatic non-sustained ventricular tachycardia, an abnormal blood pressure response to exercise and a left ventricular wall thickness > 30mm, must have an implantable defibrillator for primary prevention [19]. In 2014, a hypertrophic cardiomyopathy risk-SCD was elaborated to indicate the ICD implantation [3]. As an emerging country the sport cardiology was not developed in the sixties. Soccer players hadn´t systematically an echocardiographic imaging, some of them died on the grass, even emergency medicine was not developed. Our patient was a regional team player, he never experienced any cardiac symptom and fortunately he didn´t have any complication during his matches.

## Conclusion

Our patient had the luck to achieve his professional career as a soccer player without any rhythmic complication but in the other side he developed morbid events. Sport cardiology must be improved in our country. Even if the AHCM´s prognosis can be better all patients must have an assessment of the risk of sudden death and the family screening should be considered.
